# Preparation and Characterisation of Wood Polymer Composites Using Sustainable Raw Materials

**DOI:** 10.3390/polym14153183

**Published:** 2022-08-04

**Authors:** Satya Guha Nukala, Ing Kong, Akesh Babu Kakarla, Kim Yeow Tshai, Win Kong

**Affiliations:** 1 School of Computing, Engineering and Mathematical Sciences, La Trobe University, Bendigo, VIC 3552, Australia; 2 Department of Mechanical, Materials and Manufacturing Engineering, University of Nottingham Malaysia, Jalan Broga, Semenyih 43500, Selangor, Malaysia; 3 BASF Corporation, 1069 Biddle Avenue, Wyandotte, MI 48192, USA

**Keywords:** recycled polypropylene, sawdust, mechanical properties, thermal properties, creep analysis

## Abstract

In recent years, composites consisting of polymers and cellulosic materials have attracted increasing research attention. Polypropylene (PP) is among the most common polymer types found in excavated waste from landfills. Moreover, wood waste generated from wood products manufacturing such as sawdust (SD) offers a good potential for the fabrication of composite materials, and it is readily available in the environment. In this paper, wood polymer composites (WPC) consisting of recycled PP (rPP) and (SD) were prepared and characterised. A range of mechanical properties, including tensile strength, flexural properties, creep and hardness were studied, along with morphology, thermal properties, water degradation and contact angle. The results showed that the mechanical and thermal properties of rPP increased with an increase in 40 wt% of the SD content. Furthermore, the SD content significantly influenced the water uptake of the composites. Time–temperature superposition (TTS) was applied to predict the long-term mechanical performance from short-term accelerated creep tests at a range of elevated temperatures. The short-term creep test showed efficient homogeneity between the fillers and matrix with increasing temperature. The produced wood polymer composites displayed a comparable physical property to virgin polymer and wood and could potentially be used for various structural materials.

## 1. Introduction

Wood polymer composites (WPCs) are typically produced from the combination of two basic materials, namely wood fibres or wood flour (reinforcement) and thermoplastics (matrix) [1,2]. WPCs are frequently substituted for conventional materials owing to inherent characteristics such as low density, low processing costs, flame retardancy, mechanical properties, renewability and biodegradability. The most commonly used wood fillers are sawdust (SD) [3], wood flour [4], wood feedstocks [5] and other cellulose resources [6]. The use of these fillers as reinforcement has attracted substantial interest in the context of producing WPCs with desirable physiochemical properties for use in both structural and non-structural applications [7,8,9,10]. Additionally, wood fillers can replace synthetic fillers to reduce environmental impacts and produce eco-friendly composite products. Polyethylene terephthalate (PET) [11], polypropylene (PP) [10,12], polyethylene (PE) [10,13], polylactic acid (PLA) [14], polyvinyl chloride (PVC) [15] and polyurethane (PU) [16] are commonly used as the polymer matrices in WPCs. Among them, PP reinforced with wood fillers has been extensively studied for various industrial applications, [17,18,19,20,21,22] including construction [23], furniture [24] and automotive applications [25]. PP possesses high chemical stability, toughness and heat resistance, along with good mechanical properties. Furthermore, recycled PP (rPP) attains high performance and exhibits similar physiochemical properties to a virgin polymer matrix [17].

Englund et al. [26] studied the mechanical properties of WPCs produced using various polymer matrices such as PP, PVC and high density polyethylene (HDPE) reinforced with wood flour. The results showed that PP-based WPCs offered higher strength and stiffness along with good ductility. Moreover, the WPCs were processed repeatedly through an extrusion process, resulting in decreased voids and capillary pathways, which indicated a homogenous dispersion of wood flour in the matrix. Butylina et al. [27] reported the water absorption and mechanical properties of WPCs produced using SD-reinforced PP and SD-reinforced PLA. The results showed that PP reinforced with SD had better flexural strength and lower thickness swelling than PLA reinforced with SD. Tamrakar et al. [28] studied the water absorption and durability of wood flour–reinforced polypropylene composites prepared by extrusion. The water absorption at saturation was found to range from 16.6% to 17.0%. The tests were conducted at 21 °C for all the specimens. The obtained absorption values exceeded the limits stated in the specifications for waterfront structures by the US Army Corps of Engineers. This study also reported that the modulus of elasticity changed in response to the exposure time and temperature of the composite piles. For instance, at 0% water absorption, the modulus of elasticity was 4.3 GPa, while at 17.0% water absorption and a temperature of 21 °C, the modulus of elasticity decreased to 1.9 GPa. The study therefore confirmed that water absorption alone could degrade the modulus of elasticity of WPCs.

WPCs are exposed to various harsh environmental conditions, such as changes in temperature, humidity, and ultraviolet rays, that impact physical properties. Therefore, investigations have been carried out to evaluate the creep properties after exposure of different temperatures. Oever et al. [29] studied the creep performance of commercially available WPC profiles produced using PP matrix to determine the failure of the WPC and predict the service life of the composites used in the Netherlands. The evaluation was conducted under 50 °C at a load of 1000 N for 3 weeks. The results showed that under the moderate environmental conditions of the Netherlands, the WPCs would experience fatal failure after approximately 2 years. Tamrakar et al. [30] investigated the effect of time and temperature on the mechanical properties of the extruded wood–reinforced PP composite. By applying the time–temperature superposition (TTS) principle, long-term creep performance was predicted using short-term creep tests. The predictions revealed a lifespan of 7.9 years for the wood-reinforced PP composite.

Notably, due to growing interest in eco-friendly products, research efforts into using recycled materials in the production of WPCs have increased. Kajaks et al. [31] investigated the thermal and mechanical properties of WPCs produced from rPP and the residue from birch plywood sanding dust (PSD). The results indicated that the tensile strength of the composites increased by 25–30%, while the elastic modulus increased by 4.0–4.5 times with increase of the PSD content. Gulitah et al. [32] developed WPCs from recycled polymers and wood fibres (WF) using the compression moulding method. Different recycled polymers, such as PP, HDPE and low-density polyethylene (LDPE), were individually mixed with different ratios of WF, after which their properties were evaluated. The mechanical properties of rPP-WF at 50:50 had the highest tensile strength of 7.87 MPa, modulus of elasticity (MOE) of 520.81 MPa and modulus of rigidity (MOR) of 5.55 MPa compared to rHDPE-WF and rLDPE-WF composites, which had 5.5 MPa, 480 MPa, 3.55 MPa and 4.5 MPa, 400 MPa, 2.3 MPa, respectively. It was concluded that recycled polymer–reinforced wood fibre composites are viable alternatives to virgin polymer composites [33].

Research to date has mainly focused on the development of WPCs made of polymer waste and virgin wood fillers [34,35]. Only a limited number of mechanical performance studies have been reported for WPCs made of both recycled wood filler and polymer. Therefore, in this paper, WPCs were produced using the SD waste generated during wood product manufacturing and rPP obtained from general waste, such as disposed PP plastic containers and caps. The mechanical, thermal and water degradation effects of the produced WPCs were evaluated, and a creep analysis was conducted.

## 2. Materials and Methods

### 2.1. Materials

Recycled polypropylene (rPP) was collected from Bendigo Recycling Yard, Eaglehawk, Victoria, Australia. SD was obtained from Raw Boards Pty. Ltd., Bendigo, VIC, Australia. Sodium stearate and sodium hydroxide (NaOH) were purchased from Bunnings, Bendigo, Victoria, Australia. Hydrochloric acid (HCl) was purchased from Sigma Aldrich Pty. Ltd., Melbourne, VIC, Australia.

### 2.2. Preparation of Sawdust-Reinforced Recycled Polypropylene (rPPSD) Composites

#### 2.2.1. Cleaning of the Recycled Polypropylene (rPP)

**Figure 1:**
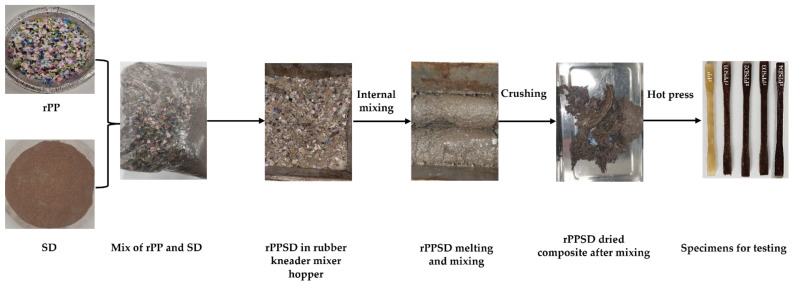
Illustration of preparation of the rPPSD composites.

The PP bottles were shredded into 8 mm pieces using a plastic crusher (Dongguan Zhongli Instrument Technology Co., Ltd., Dongguan, China) as shown in Figure 1. Subsequently, the obtained rPP pieces were cleaned with sodium hydroxide solution (5%) for 60 min. Later, the rPP was washed twice using sodium stearate, followed by water to remove excess dirt and other debris. Finally, the cleaned, shredded rPP was dried in an oven at 40 °C for 24 h to remove moisture.

#### 2.2.2. Cleaning of Sawdust (SD)

Initially, the obtained SD was sieved as per ASTM E11 standard sieve to obtain 0.05 mm. Subsequently the SD was cleaned as per the methodology reported by Medupin et al. [36]. Briefly, 25 g of SD was mixed in 150 mL of 2 M NaOH solution and stirred for 45 min using overhead mechanically stirrer at 60 rpm. The mixture was then washed three times with deionised water then filtered and mixed in 10 M HCl to remove excess alkaline. Finally, the obtained SD was washed five times with deionised water and dried in a vacuum oven at 40 °C for 24 h [36].

#### 2.2.3. Preparation of rPPSD Composites

The process of preparing the rPPSD composites is illustrated in Figure 1. Initially, the cleaned rPP and SD waste were pre-mixed in a zip lock bag according to the weight ratios (Table 1). The materials were then fed into the hopper of an internal batch mixer (ZL-3011 Rubber Lab Banbury Kneader Mixer, Dongguan Zhongli Instrument Technology Co., Ltd., Dongguan, China). The spindle speed was kept at 8 rpm and the hopper temperature was maintained constant at 190 °C throughout the mixing process. The spindle rotation direction was changed every 2 min and mixing continued for 15 min to produce the composite. Subsequently, the obtained composite was crushed into small pieces using the plastic crusher. Finally, the obtained composite fragments were hot-pressed at 190 °C with a pressure of 7 N/mm^2^ for 25 min to produce the dumbbell-shaped specimens for further characterisation. During the compression moulding process, the required thickness of the samples for further characterisation were prepared. Compression moulding shows great reproducibility and less cycle time [37,38].

### 2.3. Morphology

The morphology of the cross-sectional surfaces of the rPPSD composites was analysed via scanning electron microscope (SEM, Hitachi 3030, Tokyo, Japan). The rPPSD composites were sputter-coated with gold. The observation was performed at a voltage of 20 kV perpendicular to the cross-sectional surface.

### 2.4. Thermal Analysis

#### 2.4.1. Thermogravimetric Analysis (TGA)

The thermal properties of the samples were analysed using a thermogravimetric analyser (TGA 4000, Perkin Elmer, Waltham, MA, USA). The thermographs were obtained at thermal scan temperatures from 30 to 800 °C at 30 °C/min under a nitrogen atmosphere.

#### 2.4.2. Differential Scanning Calorimeter (DSC) Analysis

Differential scanning calorimeter (DSC 6000, Perkin Elmer, Waltham, MA, USA) was used to study the change in enthalpy of the rPPSD composites. First, the samples were heated from 30 to 190 °C at 15 °C/min. Next, the sample was held at 190 °C for 5 min to remove the thermal history, then cooled down to 30 °C at a rate of 15 °C/min to record the crystallisation rate. Subsequently, the samples were again heated from 30 to 190 °C at a rate of 15 °C/min to record the melting behaviour. To avoid oxidation in the samples, all heating and cooling runs were conducted under a nitrogen atmosphere. The percentage of crystallinity was calculated using Equation (1) [39,40]

(1) $$\%X_{c\;\;}=\frac{\Delta H_{m}}{X_{rPP}}\times \frac{100}{\Delta H_{m}^{0}}$$

where $\Delta H_{m}$ is the melting enthalpy of *rPP* or rPPSD composites, $X_{rPP}$ is the wt% *rPP* in the composite and $\Delta H_{m}^{0}$ is heat of fusion of 100% crystalline *rPP*, which is taken as 148 J/g [41].

### 2.5. Tensile Testing

The tensile testing was conducted with a universal testing machine, Zhongli ZL-8001A (Dongguan Zhongli Instrument Technology Co., Ltd., Dongguan, China) at a crosshead speed of 3 mm/min with a load cell of 500 kN. The ASTM D638 Type 4-dimension was used for the tensile testing.

### 2.6. Flexural Testing

The flexural strength of the rPPSD composites was determined using a three-point flexural test on Instron 5890 (Norwood, MA, USA) universal testing machine according to ASTM standards (ASTM D790) [42].

### 2.7. Hardness

The hardness of the composites was measured using a Vickers hardness testing machine (DuraScan G5, Kuchl, Austria). Initially, a minor load of HV 0.2 was applied before the major load of HV 0.3. The load was then applied for 10 s, and the hardness value was recorded in HV units.

### 2.8. Creep Analysis

The creep compliance at lower temperature long time equalling creep compliance at higher temperature short time was used. The master curve of the creep compliance was measured in a three-point bending mode using dynamic mechanical analysis (DMA) (DMA 8000, Perkin Elmer, Waltham, MA, USA) at a frequency of 1 Hz. The dimensions of the sample were 30 mm × 10 mm with a thickness of 4 mm. The creep compliance is given by Equation (2) [43,44]; here, *S* is the creep compliance as a function of reference temperature (*T_ref_*) and time (*t*), *T_elev_* is the elevated temperature and $\propto_{T}$ is the shift factor. Creep and creep recovery cycles were conducted at isotherms between 20 and 70 °C at intervals of 5 °C. For each isotherm, 20% of the average flexural strength was applied for 1 h, followed by a 1 h recovery period.

(2) STref , t=STelev, t∝T

### 2.9. Soil Burial Degradation

The rPPSD composites were buried at a depth of 2 cm in a mixture of 50% sand and 50% black humus soil at ambient temperature [45,46]. The relative humidity of the soil was about 50–60%. The degradation of the composites was assessed at predetermined intervals of 4, 8, 12, 16 and 20 days, respectively. Before weighing, the composites were removed from the soil and washed with distilled water. The weight loss of the composites buried in soil was obtained using Equation (3) [45,46]. Here, *M*_1_ and *M*_2_ represent the weight of the composites before and after soil burial, respectively.

(3) $$Weight\;loss\;\left(\%\right)=\frac{M_{2}-M_{1}}{M_{1}}\times 100$$

### 2.10. Water Absorption

Samples with a dimension 30 × 30 × 3 mm^3^ were used for the water absorption analysis. The samples were dried in an oven for 24 h at 80 °C to obtain constant weight. Subsequently, the initial weight (*W*_1_) was recorded. The samples were then immersed in water for 2, 4, 6, 8, 10, 12, 14, 16, 18 and 20 days, respectively. The weight was recorded at regular time intervals to obtain the water absorption (%). The water absorption of the sample was calculated using Equation (4) [47].

(4) $$Water\;absorption\;\left(\%\right)=\left[\left(W_{2}-W_{1}\right)/W_{1}\right]\times 100$$

where *W*_1_ and *W*_2_ are the sample weights before and after immersion, respectively.

### 2.11. Water Contact Angle

The wettability of the composites was measured using the Attension Theta Flux (Biolin Scientific, Västra Frölunda, Sweden). The contact angle measurement was conducted via sessile drop technique. The droplet was placed on the surface of the samples using a micrometre syringe, and the contact angle was measured by scanning the droplet profile for 15 s [48,49]. To avoid the effects of weight, the size of the water droplet was maintained at about 2 μL [50].

### 2.12. Statistical Analyses

GraphPad Prism 9.0 (GraphPad Software, Inc., San Diego, CA, USA) was used to evaluate statistical analyses using the ANOVA method. Three samples (*n* = 3) were investigated for each data set and presented as mean ± standard deviation (SD) unless otherwise stated. A significance level of the *p*-value of ≤0.05 was determined to be significant (*). Error bars in all figures represent the standard error of the mean [51,52,53].

## 3. Results and Discussion

### 3.1. Morphology

**Figure 2:**
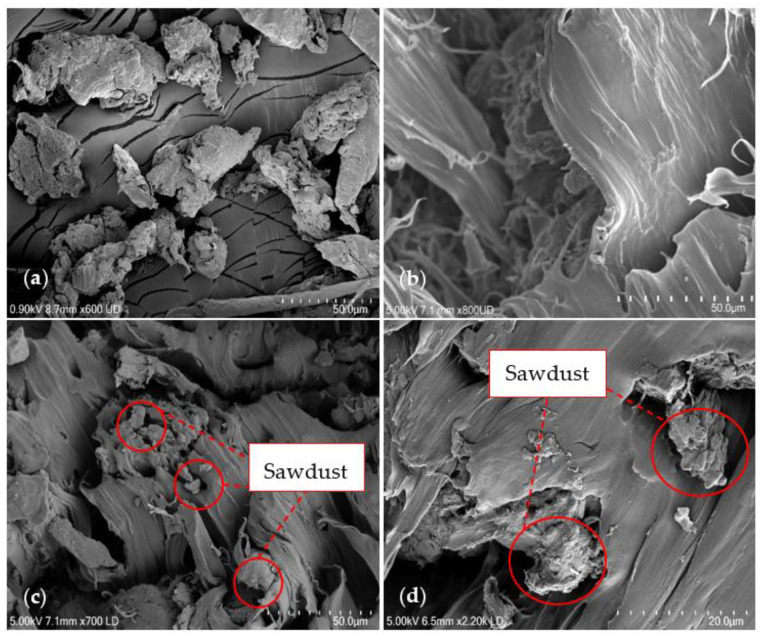
SEM Micrographs of (**a**) SD; (**b**) rPP; (**c**) rPPSD4 at low magnification; and (**d**) rPPSD4 at high magnification.

The properties of the rPPSD composites are highly dependent on the dispersion of SD in the matrix and the interaction between the SD and the polymer matrix. Therefore, the morphology of the SD, rPP and rPPSD composites was observed to provide further insight into the microstructures. Figure 2a shows the micrograph of the SD. As is evident from the image, SD was loose and rough. The roughness of the SD was further evident in the composites fabricated with rPP. Figure 2b shows the cross-sectional SEM image of the rPP, which was smooth and featureless [40]. The strength and interfacial interactions determined the composites failure mode and micromechanical deformation, as reported by Renner et al. [54]. Figure 2c,d shows the morphology of rPPSD4 composites at lower and higher magnification micrographs. The red circles highlighted the SD embedded in the polymer matrix, suggesting good interfacial adhesion between SD and polymer matrix, along with uniform dispersion of SD throughout the cross-section of the compressed samples [6,40].

### 3.2. Thermal Properties

#### 3.2.1. Thermogravimetric Analysis (TGA)

**Figure 3:**
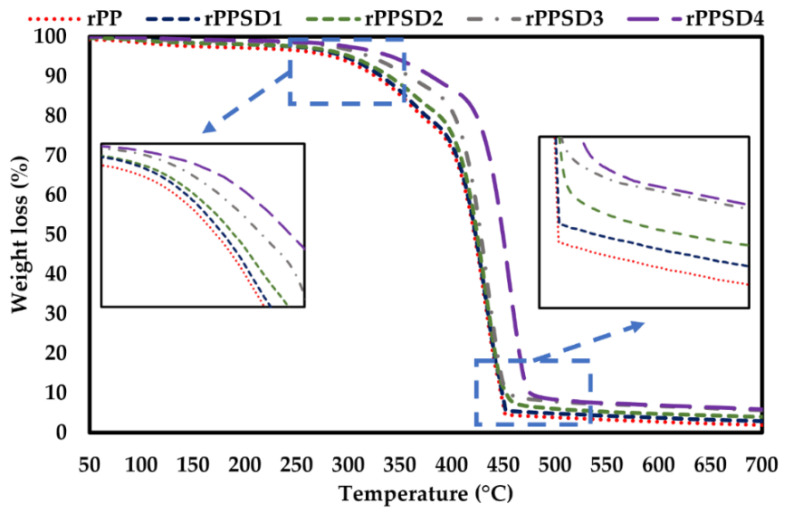
TGA curves of rPPSD composites.

**Figure 4:**
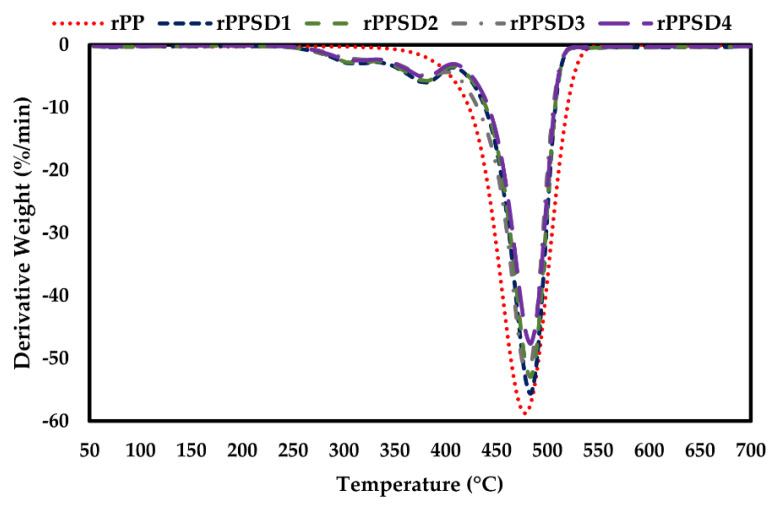
DTG curves of rPPSD composites.

The TGA and DTG curves for the rPPSD composites are shown in Figure 3 and Figure 4, while the thermal properties are shown in Table 2. The weight loss occurring between 70 and 110 °C was associated with the evaporation of absorbed moisture from the samples. In general, all the composites were thermally stable up to 200 °C. From the TGA curves, it is evident that hemicellulose started its degradation reactions followed by the more thermally stable cellulose domains at 250 °C [55,56,57]. Degradation of cellulose and hemicellulose involved complex reactions comprised in the temperature range of 250–370 °C. The degradation of lignin occurred in a wider temperature range from 250–480 °C. The addition of SD into the rPP matrix enhanced the thermal stability of composites. As shown in Table 2, the onset temperature progressively increased from 255 °C for rPP to 350 °C for rPPSD4. A similar trend can be observed in the end set temperatures of the rPPSD composites where the temperature increased from 452 °C (rPP) to 470 °C (rPPSD4). The contents at 700 °C are residual char or ashes from decomposition of saw dust. The increase in on set and end set temperature with increasing SD composition implied that SD enhanced thermal insulation behaviour of rPP matrix. According to Chun et al. [58] and Zander et al. [59], the rPP reinforced with cellulose fibres displayed improved thermal stability with an increase in fibre content. Similar observations were made with other types of WPCs [8,12,60,61,62,63,64,65,66].

#### 3.2.2. Differential Scanning Calorimetric Analysis (DSC)

**Figure 5:**
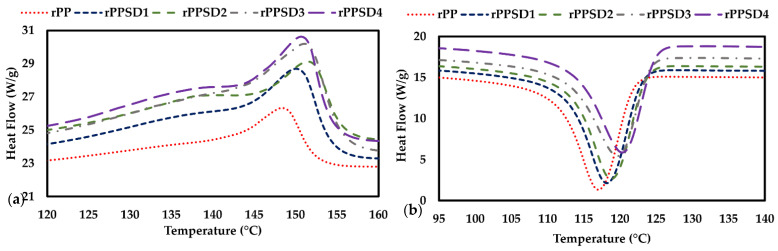
(**a**) Melting and (**b**) crystallisation curves.

The DSC curves of rPPSD composites are shown in Figure 5. The values of T_m_ (melting temperature), T_c_ (crystallisation temperature), ΔH_c_ (crystallisation enthalpy), ΔH_m_ (melting enthalpy) and X_c_ (crystallinity; %) are tabulated in Table 3. The T_c_ increased from 117.1 °C to 120.9 °C as the weight of SD in rPP increased from 10 to 40 wt% [67]. The primary mechanism responsible for the crystallisation and melting behaviour of the matrix is heterogeneous nucleation on the SD surfaces [68,69]. Therefore, the increase in T_c_ and T_m_ values of the rPPSD composites was due to the presence of SD which acts as a nucleating agent [70,71,72].

The values of ΔH_c_ (crystallisation enthalpy) and ΔH_m_ (melting enthalpy) for rPP were 95.0 and 93.7 J/g, respectively. The ΔH_c_ and ΔH_m_ were significantly increased with the increase in SD reinforcement into rPP. Ndiaye et al. [40] stated that the polymer in the wood polymer composite formed crystals much more easily due to the nucleation effect of the wood particles with the addition of lower content of wood particles. According to Lee et al. [73], more heat energy was absorbed by wood flour in melting the WPC. The X_c_ of the rPPSD composites increased with the increase in SD incorporation. The X_c_ for rPPSD1, rPPSD2, rPPSD3 and rPPSD4 were 56.5%, 57.7%, 58.5% and 59.8%, respectively. Beg et al. [74] reported a 3.3% increase of X_c_ with 40 wt% of wood flour in PP. Similar observations were made in the literature [73,74,75,76,77].

### 3.3. Tensile Properties

**Figure 6:**
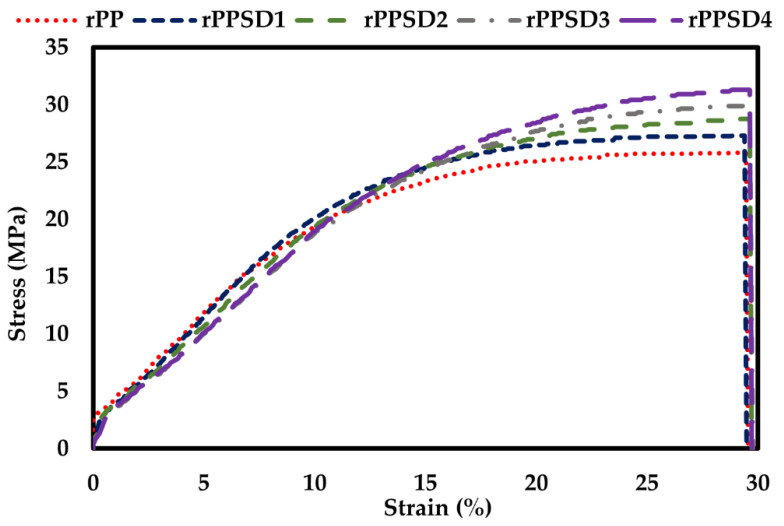
Tensile stress–strain curves of rPPSD composites.

The tensile performance of the rPPSD composites is shown in Figure 6. The rPP presented a tensile strength of 25.5 MPa and Young’s modulus of 3.79 MPa. The incorporation of SD increased the ultimate tensile strength and Young’s modulus with rPPSD4 showed the highest ultimate tensile strength and Young’s modulus of 31 MPa and 6.95 MPa, as shown in Table 4. Ndiaye et al. [78] reported that the highest tensile strength was found in 70% PP with 30% wood composite. Najafi et al. [79] reported similar results with WPCs made of recycled plastics, where the stress concentration increased with an increase in the wood content. As shown in the SEM micrographs (Figure 2a–d), there was no clear gap between the SD and rPP matrix, showing a good interface bonding and indicating the stress transfer from the weaker matrix to the strong wood fibre.

### 3.4. Flexural Properties

**Figure 7:**
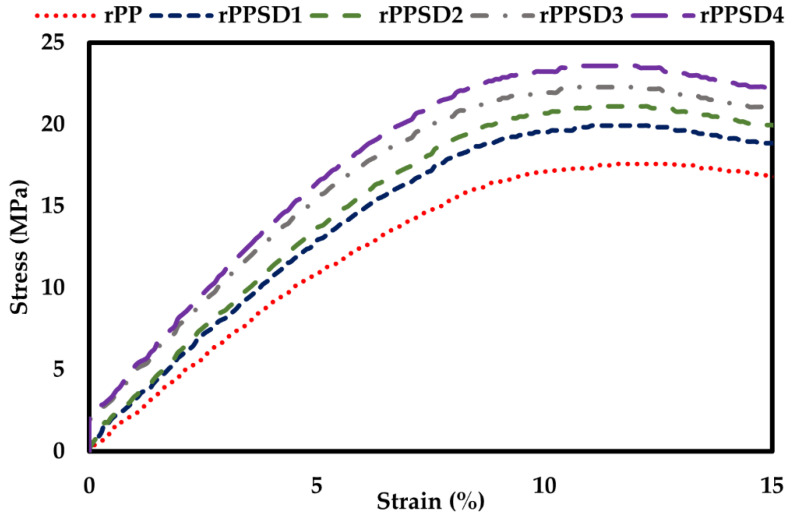
Flexural stress–strain curves of rPPSD composites.

The flexural behaviour of the rPPSD composites is shown in Figure 7. The flexural strength of the rPPSD composites increased linearly with the increase of SD content. The rPP showed the lowest flexural strength at 17 MPa, followed by rPPSD1, rPPSD2 and rPPSD3 with 19.5, 20.5 and 21.5 MPa, respectively. The rPPSD4 showed the highest flexural strength among all composites, at 23 MPa. The failure of specimens initiated with a crack on the tension side and grew until complete failure. Ratanawilai et al. [80] reported that when PP was mixed with wood flour at different concentrations, the highest flexural strength was found at 60% PP and 40% wood content. Furthermore, uniform dispersion and SD content play a significant role in determining the flexural properties of WPCs [78].

### 3.5. Hardness

**Figure 8:**
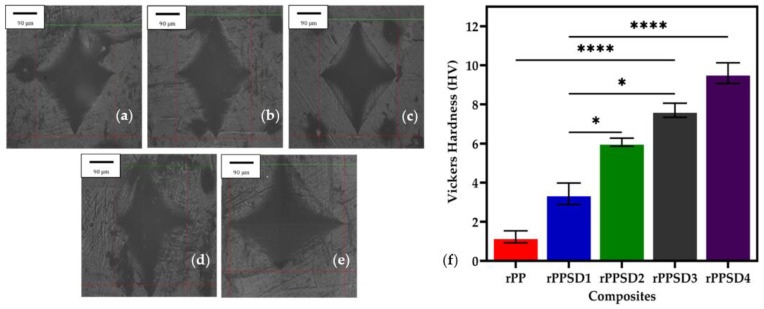
Optical images of Vickers microhardness indentation on (**a**) rPP; (**b**) rPPSD1; (**c**) rPPSD2; (**d**) rPPSD3; (**e**) rPPSD4; and (**f**) Vickers hardness number of rPPSD composites (*n* = 3, * *p* ≤ 0.05, **** *p* ≤ 0.0001).

The optical micrographs of the microhardness indentation are shown in Figure 8a–e and hardness values of the composites are shown in Figure 8f. A continuous increase in hardness values was observed as the concentration of SD increased in the rPPSD composites [81]. This increase in hardness can be attributed to the fact that the addition of SD in rPP imparted stiffness, thus making the composites rigid and hard and restricting the mobility of polymeric chains [82,83]. The rPP had a hardness of 1.5 HV, whereas the composites had a hardness of 3.7 HV (rPPSD1), 6.3 HV (rPPSD2), 7.8 HV (rPPSD3) and 9.8 HV (rPPSD4). The present results agreed with the prior literature [78,84,85].

### 3.6. Creep Analysis

**Figure 9:**
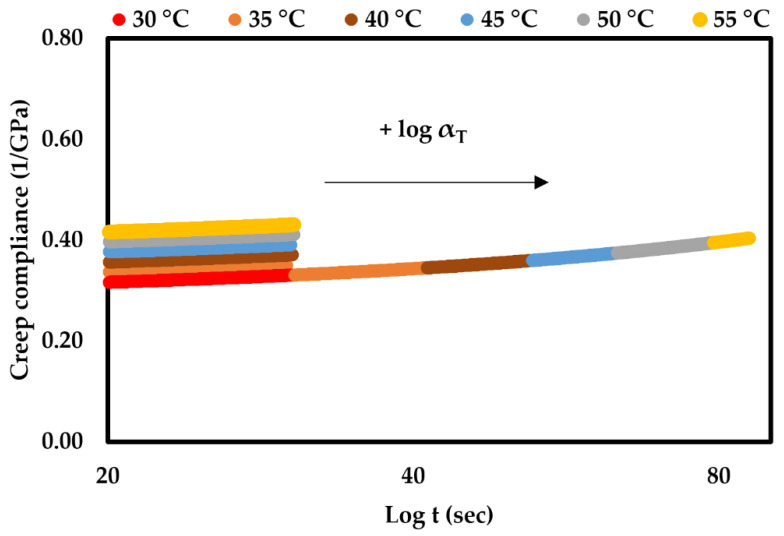
Unshifted creep compliance and corresponding master curves of rPPSD4.

The creep analysis was performed using TTS to predict the long-term creep behaviour of the rPPSD composites from short-term accelerated creep tests at a range of elevated temperatures. rPPSD4 was taken as the measuring sample. Figure 9 presents the unshifted short-term creep compliance and corresponding master curves of rPPSD4 at all tested temperatures, which were plotted against the test time on a log scale. With time and temperature increases, the creep compliance increased due to composite becoming less stable as the magnitude of creep strain increased over the same period of loading, which affects the viscoelastic region of the composite. For example, the creep compliance increased from 0.35 1/GPa at 30 °C to 0.44 1/GPa at 55 °C as the composite experienced greater deformation due to the constant applied stress. The shift factor was calculated using the modified William–Landel–Ferry (WLF) equation.

For the rPPSD4 composite, the modified WLF equation was employed as presented by Nielsen et al. [86] (see Equation (5) below) to calculate the shift factor when a temperature other than *T_g_* is chosen as the reference temperature.

(5) $$\mathrm{Log}\left(\propto_{T}\right)=\frac{-C_{1}{}_{}\left(T-T_{ref}\right)}{C_{2}+\left(T-T_{ref}\right)}$$

Here, $\propto_{T}$ is the horizontal shift factor for the corresponding elevated temperature, *T* (°C); the reference temperature is *T_ref_* (°C); and finally, $C_{1}$ and $C_{2}$ are the empirical constants determined from Equations (6) and (7).

(6) $$C_{1}=\frac{C_{1g}C_{2g}}{C_{2g}+T_{ref\;}-T_{g}}$$

(7) $$C_{2}=C_{2g}+T_{ref\;}-T_{g}$$

In the above, $C_{1g}\;$and $C_{2g}$ are the empirical constants ($C_{1g}$ = 17.44 and $C_{2g}$ = 51.6 °C), while $T_{g}$ is the glass transition temperature (°C).

By substituting the $T_{g}\;$(°C) of WPC materials into Equations (6) and (7), the values of $C_{1}$ and $C_{2}$ were calculated to be 10.5 and 85.70 °C, respectively. Finally, by substituting these values in Equation (5), the shift factor for the rPPSD4 composite was calculated to be $4.025\times 10^{-3}$ for 45 °C [30,86,87].

### 3.7. Soil Burial Degradation

**Figure 10:**
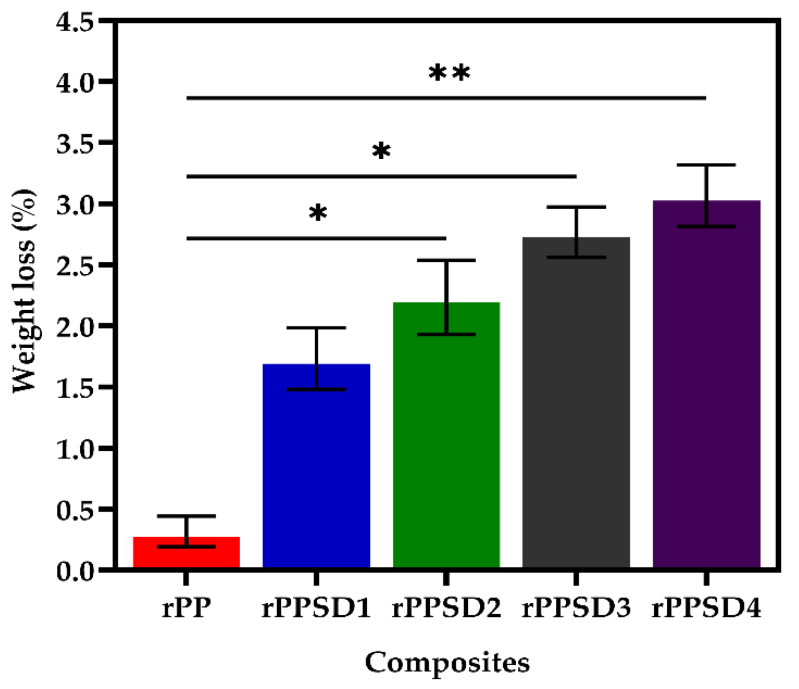
Soil degradation of rPPSD composites (*n* = 3, * *p* ≤ 0.05, ** *p* ≤ 0.01).

Soil burial degradation occurs due to moisture and enzymatic action in soil, leading to weight loss in the material [88,89]. Figure 10 shows the weight loss of each composite after being subjected to soil burial for 20 days. The weight loss percentage are 0.45% for rPP, 1.7% for rPPSD1, 2.3% for rPPSD2, 2.8% for rPPSD3 and 3.3% for rPPSD4. With the increase in wt% of SD, the weight loss percentage for soil burial degradation of rPPSD composites increased. The chemical contents such as cellulose, hemicellulose and lignin presented in SD reacted with soil and caused weight loss of the composite through soil burial [45,46]. The hydrolysis of the polymer backbone was the primary reason for the degradation of rPP. The increased water absorption (as depicted in Section 3.8) in composites with higher wt% of SD leads to more pronounced hydrolysis compared to rPP. Further, with the help of the moisture in the soil, the polymer chains were demolished by creating tiny fragments of rPPSD composite [46]. Finally, the microbial activities promoted the weight loss of the composite during soil burial degradation [89,90].

The study conducted by Yang et al. [46] on bamboo fibre–reinforced PP composites stated that with the increase in soil burial time and filler concentration, the weight loss of composites increased. The present study aligns with the study of Yang et al. [46].

### 3.8. Water Absorption

**Figure 11:**
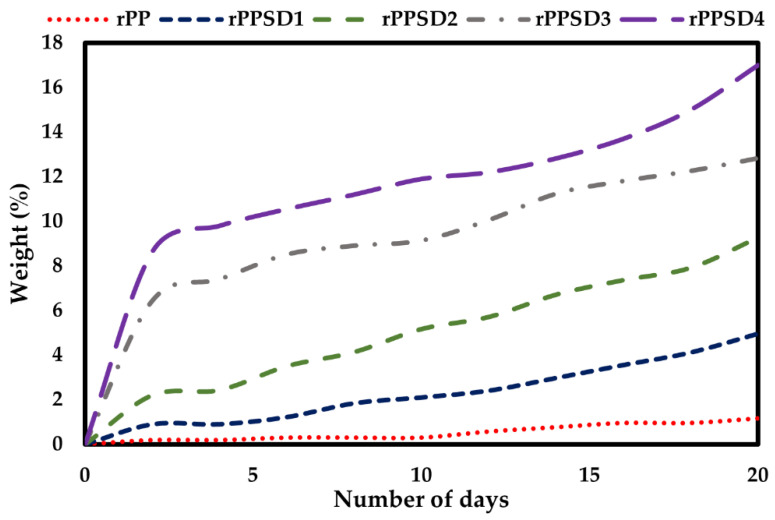
Water absorption curves of rPPSD composites.

The water absorption behaviour of the rPPSD composites measured for 20 days with measurements taken at regular intervals is shown in Figure 11. The water absorption increased quickly in the first 3 days, and it slowed down as the immersion time prolonged until the specimen’s water content was saturated [91,92]. When the samples were in an equilibrium state of water absorption (allowing the water absorption to change in time range and period of immersion), the percentages of water absorption of rPP, rPPSD1, rPPSD2, rPPSD3 and rPPSD4 were 1.15%, 4.95%, 9.25%, 12.82% and 17%, respectively. The rPP showed a low absorption percentage as it was hydrophobic and thus absorbed very little water [93]. The composites showed similar water absorption curves for the entire immersion period. Therefore, the water absorption percentages were considered to be consistent with Fickian diffusion [94,95,96]. Moreover, it shows that SD played a profound role on the water absorption behaviour of samples, that is, the water absorption increased with the increase of SD content, which is also consistent with the studies reported by other researchers [6,97,98,99,100,101,102].

### 3.9. Water Contact Angle

**Figure 12:**
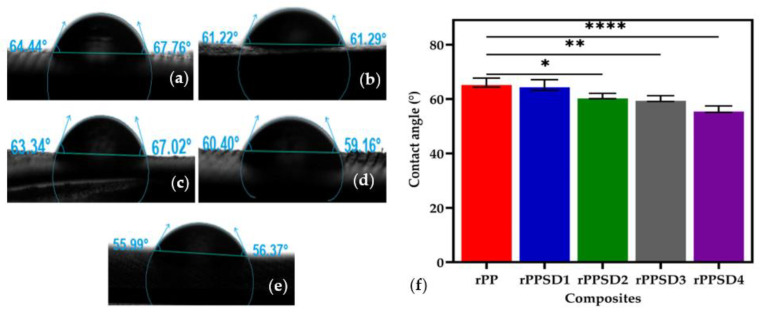
Contact angle and sessile drop images of (**a**) rPP; (**b**) rPPSD1; (**c**) rPPSD2; (**d**) rPPSD3; (**e**) rPPSD4; and (**f**) contact angle values of the PPSD composites (*n* = 3, * *p* ≤ 0.05, ** *p* ≤ 0.01, **** *p* ≤ 0.0001).

The water contact angle was measured to investigate the hydrophilicity of the rPPSD composites, as shown in Figure 12. According to the literature, a contact angle below 90° indicates a good wetting surface by any liquid [53,103,104,105]. The measurements of water contact were in accordance to those reported by Sdrobiş et al. [48] and Wang et al. [49]. Figure 12 shows the sessile drop images and the contact angle measurements of the composite surfaces. The rPP possessed the highest contact angle, with 66.1°, compared to the rPPSD composites as it was hydrophobic in nature, thus absorbing less water [106]. On the other hand, composites with the incorporation of SD showed a decrease in water contact angle, attributed to the hydrophilic nature of SD [107]. The average contact angles of rPPSD1, rPPSD2, rPPSD3 and rPPSD4 were 65.18°, 61.25°, 59.78° and 56.18°, respectively. Similarly, Lazrak et al. [108] studied the wetting behaviour of wood flour reinforced rHDPE composites, and it was stated that a decrease in contact angle can be attributed to an increase in wood flour, which is hydrophilic in nature. The present study aligns with the literature mentioned above.

## 4. Conclusions

This study successfully fabricated rPPSD composites using an internal batch mixer and compression moulding technique. The morphology, mechanical, thermal, soil burial degradation, water absorption and wettability properties of the produced rPPSD composites with varied SD and rPP contents were analysed. The morphological images showed that SD dispersed in the rPP matrix uniformly. The thermal properties showed that increased content of SD in the rPP matrix improved the thermal stability of rPPSD composites. Furthermore, the tensile and flexural strength increased from 25.5 MPa to 31 MPa and 18 MPa to 24 MPa for 40 wt% of SD in rPP, respectively. The creep compliance for rPPSD4 was increased from 0.35 1/GPa at 30 °C to 0.44 1/GPa at 50 °C as the composite experienced greater deformation under constant applied stress. In addition, the soil burial degradation showed a considerable weight loss, up to 3.3%, with the increase in SD content. Similarly, the water absorption increased with the increase in the SD concentration, while the wettability increased in rPPSD composite with higher concentrations of SD; for example, the water contact angle decreased and hydrophilicity increased. Hence, the results indicated that the properties and the performances of the rPPSD are similar or comparable to composites made of virgin wood and plastics. The rPPSD could potentially be used as an alternative material to replace non-sustainable composites.

## Figures and Tables

**Table 1:** Composition of rPPSD composites.

Sample Name	rPP (wt%)	SD (wt%)
rPP	100	0
rPPSD1	90	10
rPPSD2	80	20
rPPSD3	70	30
rPPSD4	60	40

**Table 2:** Thermal properties of rPPSD composites, obtained from TGA.

Composites	On Set Temperature (°C)	End Set Temperature (°C)	Residual Weight (%)
rPP	295	452	0.9
rPPSD1	305	453	2.1
rPPSD2	310	456	2.5
rPPSD3	330	457	2.6
rPPSD4	350	470	2.7

**Table 3:** Thermal properties of rPPSD composites from DSC.

Composites	T_m_ (°C)	T_c_ (°C)	ΔH_m_ (J/g)	ΔH_c_ (J/g)	X_c_ (%)
rPP	148.6	117.1	93.7	95.0	45.6
rPPSD1	149.9	118.4	102.6	105.3	56.5
rPPSD2	150.8	119.2	109.8	105.6	57.7
rPPSD3	151.5	120.3	112.3	110.0	58.5
rPPSD4	151.6	120.9	115.6	110.3	59.8

**Table 4:** Mechanical characteristics of rPPSD composites.

Composite	Young’s Modulus (MPa)	Ultimate Tensile Strength (MPa)
rPP	3.79 ± 0.10	25.5 ± 0.1
rPPSD1	4.12 ± 0.20	26.8 ± 0.1
rPPSD2	5.72 ± 0.20	27.4 ± 0.1
rPPSD3	5.34 ± 0.15	28.1 ± 0.5
rPPSD4	6.95 ± 0.15	31 ± 0.5

## Data Availability

Not applicable.
